# Hope in Every Breath: Navigating the Therapeutic Landscape of Cystic Fibrosis

**DOI:** 10.7759/cureus.43603

**Published:** 2023-08-16

**Authors:** Himabindu Sreenivasulu, Sudheer kumar Muppalla, Sravya Vuppalapati, Mohammad Shokrolahi, Apeksha Reddy Pulliahgaru

**Affiliations:** 1 General Medicine, People's Education Society (PES) Institute of Medical Sciences and Research, Kuppam, IND; 2 Pediatrics, People's Education Society (PES) Institute of Medical Sciences and Research, Kuppam, IND; 3 Internal Medicine, University of Debrecen, Debrecen, HUN

**Keywords:** pulmonary screening, patient education, respiratory care, gene therapy, chest physiotherapy, hand hygiene compliance, infection prevention and control, cftr modulating therapy, cystic fibrosis (cf)

## Abstract

Cystic fibrosis (CF) has long posed a complex challenge to medical science. Still, the tides are turning with remarkable progress in prognosis and demographics, thanks to cutting-edge medical management and treatment breakthroughs. It affects multiple systems, necessitating a comprehensive approach to its management. This article thoroughly reviews the latest advancements in CF treatment across three key areas: respiratory care, infection prevention, and pharmacological management. In respiratory care, emphasis is placed on airway clearance therapies and nebulized saline, while infection prevention strategies encompass hand hygiene, respiratory etiquette, and environmental cleaning and disinfection. Pharmacological management explores pancreatic enzyme replacement therapy (PERT), antimicrobial treatments, cystic fibrosis transmembrane regulator (CFTR) modulators, and promising gene therapies. Patient education and support are highlighted as crucial components of effective CF management, while mental health assessments are emphasized due to CF patients' susceptibility to anxiety and depression. This review highlights the tremendous progress made in the management of CF. Integrating early detection, infection prevention, pharmacological interventions, gene therapy, and patient support is revolutionizing the care and quality of life for individuals with CF.

## Introduction and background

Within the realm of genetic puzzles, cystic fibrosis (CF) emerges as a fascinating multisystemic and chronic disease with an autosomal recessive pattern of inheritance, where pathogenic mutations in the cystic fibrosis transmembrane regulator (CFTR) gene on chromosome 7 (locus 7q.31) play a pivotal role [1]. The historical depiction of CF as the most prevalent life-threatening inherited condition among Caucasian children, with an incidence of 1 in 2,500 live births, no longer holds in contemporary times [2]. Since its initial description in 1938, CF has witnessed a remarkable shift in prognosis and demographics [2]. Once a condition where patients rarely survived beyond their first year, CF has now transformed into an adult disease, with adults outnumbering children in developed nations and a projected median survival age approaching 50 years, signifying a significant improvement where half of the newborns diagnosed with CF today can anticipate living well into their fifth decade, accompanied by new challenges in managing associated conditions [2]. Early diagnosis, a better understanding of the disease's natural course, and better treatments, including aggressive nutritional support, improved mucociliary clearance and mucus drainage, prompt antimicrobial and anti-inflammatory therapy, timely treatment of pulmonary exacerbations, infection control measures inside and outside CF centers, and early detection and management of CF-related complications, have all contributed to this increase in survival [3]. In a pivotal moment 25 years ago, the discovery of the CFTR gene illuminated the path toward unlocking the intricate secrets of CF lung disease, propelling us into an era of molecular and cellular exploration [4]. While CF continues to be a severe disorder, it is crucial to recognize the transformative impact of substantial advancements in medical management and treatment, which have reshaped the demographic landscape of the patient population in remarkable ways [2].

## Review

Screening and diagnosis

Since 2010, universal CF screening has been available in all 50 states of the United States, changing the picture of CF diagnosis significantly. Most nations with high CF incidence have also instituted universal neonatal CF screening [5]. The following tests are regularly used for screening and diagnosis.

Sweat Chloride Test

Measuring chloride concentration in sweat is the most trustworthy and accessible diagnostic test for CF [6]. A sweat test must also be used to confirm CF diagnosis in newborns with a positive newborn screening (NBS) test [7].

The sweat chloride test continues to be the gold standard for diagnosis since it evaluates CF transmembrane regulator activity directly. Skill and expertise are necessary for the sweat chloride test to be performed correctly, which is essential for a precise diagnosis of CF [8]. Pilocarpine is applied transdermally through iontophoresis to promote sweat gland production, after which perspiration is collected in a Macroduct coil, gauze, or filter paper, and the chloride content is assessed. A CF diagnosis is consistent with a sweat chloride level of more than or equivalent to 60 mmol/L [9].

Cystic Fibrosis Transmembrane Regulator Gene Mutations

The CF transmembrane conductance regulator, which regulates the movement of chloride ions across epithelial cell membranes in the pancreas, stomach, and lungs, is the root cause of the disease [10]. As a result of poor or nonexistent chloride transport, mucus builds up in CF patients, increasing the risk of infections and blockages. The likelihood of dehydration and mineral imbalances rises when perspiration contains excessive chloride. Although there are several additional connected issues, these characteristics of the condition often result in poor nutrition and development, recurrent respiratory infections, and lung damage [11].

Identifying the mutations causing CF in the specific patient is crucial since medicines targeted at particular mutations are now accessible. This is accomplished by first performing a conventional CFTR mutation panel, which covers 80-85% of all variants in the population and includes the most prevalent disease-causing mutations in the population under study. The second stage is thorough sequencing of the whole CFTR gene together with evaluation of significant deletions or insertions when two CFTR mutations are not found, and the diagnosis of CF is almost definite (sweat chloride concentration >60 mmol/L) or highly probable (e.g., a suggestive clinical picture) [6].

Over 30 years have passed since the CFTR gene was identified. A total of 90% of CF patients had one or both alleles of the most prevalent CFTR mutation, deletion of the phenylalanine residue at position 508 (Delta F508) [6,12]. The loss-of-function phenotype is caused by almost 2000 additional mutations that either prohibit full-length translation or affect the folding, stability, and channel gating of the CFTR [13,14].

Nasal Potential Difference Measurement

The Nasal potential difference (NPD) is a bioassay for CFTR function, similar to the sweat test. NPD is exceptionally specialized and not publicly accessible. NPD tests are now standardized and have demonstrated their value in confirming or disconfirming CFTR impairment [15,16]. The transmucosal voltage potential is measured using the NPD, an in vivo test. The nasal mucosa in CF absorbs more salt and secretes less chloride than the healthy nasal mucosa.

This is demonstrated by a higher negative baseline nasal potential in CF patients compared to healthy controls, a more pronounced shift in potential with sodium channel blockade, and little to no change in potential upon activation of the CFTR channel by isoproterenol and a zero chloride solution [6]. The epithelial Na+ channel (ENaC) function is represented by the first two values of the NPD measurement, and the CFTR channel function by the last two. The NPD values show a gradient of abnormalities, from CF patients with inadequate pancreas through CF patients with sufficient pancreas to cystic fibrosis transmembrane regulator-related disorders (CFTR-RD) heterozygotes and healthy individuals [17].

Diagnostic Criteria

Positive results on neonatal screening or CF-suggestive signs and symptoms or a parent or sibling with a good family history. Additionally, either a sweat chloride level greater than or equivalent to 60 mmol/L, the discovery of two trans mutations that cause CF, or an NPD measurement that is compatible with CF [5].

Nutrition

The Cystic Fibrosis Foundation (CFF) provides general nutritional guidelines for individuals with CF, which also apply to those with CFRD with slight modifications. Due to increased energy expenditure and malabsorption, most CF patients require a high caloric intake to maintain weight, with recommendations ranging from 110% to 200% of the daily recommended caloric intake for the general population [18]. 

Corey et al. reported differences in patient status between Boston and Toronto, with the US recommending a low-fat diet. At the same time, Toronto did not, but recent comparisons suggest that higher lipid intake may have a more substantial impact than just high energy intake in CF patients [19]. Although general guidelines recommend high energy intake in CF patients, achieving the recommended fat intake is often challenging. It is essential to shift the focus toward the type of fat consumed, emphasizing linoleic-rich oils like sunflower oil and corn oil, as well as encouraging fish or omega-3 supplementation to mitigate inflammation, which could serve as beneficial additions to CF modulator therapy while awaiting further research [19]. Routine supplementation of vitamins K and E is supported by satisfactory evidence in individuals with CF. In contrast, individualized supplementation guided by biochemical assessment and previous response is recommended for vitamins A and D due to the need for more evidence for routine supplementation [20]. 

Individuals with CF are at an increased risk of iron deficiency due to various factors, including chronic inflammation, inadequate dietary intake, and gastrointestinal issues, and the guidelines recommend regular assessment of iron status using specific markers and interpretation of results. Similarly, people with CF are prone to sodium deficiency, primarily due to the CFTR gene defect affecting sodium and chloride ion transport, and the guidelines provide recommendations for sodium intake and strategies for sodium supplementation based on individual needs and environmental factors, such as hot and humid climates [20]. While the CFF has not formally updated its CF Nutrition Guidelines to address the emerging challenges of overweight and obesity and the availability of CFTR modulators, recent evidence-based guidelines from other organizations emphasize the importance of individualized nutrition care based on genetic mutations, clinical status, and personal health goals [21]. These guidelines recommend a diet that aligns with general population recommendations, focusing on improving diet quality and addressing specific needs such as calorie reduction and additional nutrient benefits [21]. 

Physiotherapy

While oscillatory devices show potential in clearing secretions and improving sputum volume, no statistically significant evidence indicates their superiority over other physiotherapy techniques regarding short-term respiratory function outcomes [22]. Although inspiratory muscle training and chest physiotherapy failed to improve other performance metrics, except for maximum inspiratory pressure, a comprehensive chest physiotherapy program appears to be an individual effective method for improving dynamic postural stability, spirometry, respiratory muscle strength, and six-minute walk distance (6MWD) on the limits-of-stability test (LOST) [23]. 

The Cochrane review also highlights the strong evidence that positive expiratory pressure (PEP) with a mask is more effective at reducing pulmonary exacerbations than active cycle breathing techniques, oral oscillating PEP devices, autogenic drainage (AD), high-frequency chest wall oscillation (HFCWO), BiPAP, and exercise, highlighting the need for personalized airway clearance strategies based on developmental phases, pulmonary symptoms, patient preferences, and lung function by accounting for changes in baseline function and aggravating factors [24]. The evidence from four randomized controlled trials evaluating exercise interventions in individuals who suffer from CF suggests very low-certainty evidence regarding the effects on adherence, lung function, and other outcomes, highlighting the need for further research [25]. 

Infection prevention

To reduce the risk of respiratory infections and protect the health of people with CF, it is essential to prevent infectious illnesses. To stop the transmission of pathogens, stringent cleanliness practices and adherence to infection control guidelines are necessary [26].

Maintaining Strict Hand Hygiene

Handwashing is a fundamental and highly effective practice for infection control among individuals suffering from CF. According to a study conducted by Zuckerman et al., which included 97 participants from seven study sites, the results confirmed that "the use of alcohol-based hand hygiene products significantly reduced the presence of respiratory pathogens on hands." Additionally, they found that frequent hand hygiene during office visits is necessary to minimize the risk of recurrent contamination [26]. 

Adhering to Respiratory Hygiene

Wood et al. evaluated the efficacy of different face masks and cough etiquette (covering mouth with hand while coughing) in lowering cough aerosols of physician assistants created by persons with CF in a laboratory cough rig. Face masks minimize *Pseudomonas aeruginosa* aerosols produced by coughing, with the surgical mask giving further comfort. Coughing etiquette had a lower effect in reducing viable aerosols [27]. Zuckerman et al. investigated room contamination rates between patients wearing surgical masks and those who did not and discovered that the two groups had equal contamination rates [28]. The Stockwell et al. studies support the recommendation by US infection control guidelines for CF that states CF sufferers should wear masks when at a medical facility [29]. It has recently been suggested that masks should only be used in public spaces and not, for example, in the patient's private side room [30].

Zuckerman et al. discovered that air contamination with CF respiratory infections was rare during clinic visits, and surgical masks did not minimize contamination in exam rooms [31]. He found that the spirometry room's air was more likely to be contaminated by creating contaminated aerosols during forced expiratory movements and coughing. This is corroborated by a tendency toward higher cough frequency with spirometry testing during air pollution interactions. By 30 minutes, the contamination had disappeared.

Participants' age, particularly CF infections, signs and symptoms of pulmonary exacerbations, and air exchange rates were unrelated to air pollution. His findings support the updated Infection Prevention and Control Guideline (IP&C) for CF patients recommending a minimum of 30 minutes elapse between patients with CF while undergoing spirometry; this recommendation can be neglected if negative pressure ventilation or high-efficiency particulate absolute filtration is used [31].

Cleaning and Disinfection of the Environment

To mitigate the spread of healthcare-associated pathogens, it is essential to implement improved cleaning and disinfection strategies in patient rooms post-discharge, a process commonly known as terminal cleaning; alongside this, a daily disinfection routine should be established for high-touch surfaces within isolation rooms to prevent potential contamination. Moreover, it is crucial to disinfect portable medical equipment between patient use or opt for disposable or dedicated equipment exclusively for isolation rooms. These proactive measures are pivotal in reducing cross-contamination and enhancing overall infection control efforts in healthcare settings [32,33].

Patient and Provider Education

The CFF recommends providing CF-specific IP&C education for individuals diagnosed with CF and their families. This education should accommodate the target audience's age, language proficiency, and reading abilities. It is crucial to involve CF patients and their families in developing educational programs and implementing recommended practices. The education should be periodically reinforced as determined by each healthcare facility [29]. Similarly, healthcare staff responsible for caring for individuals with CF should receive education on CF-specific IP&C. Adult learning principles should be applied to ensure effective knowledge transfer, and each healthcare center should determine the frequency of these educational sessions [29].

Equipment Handling

In a study by Jakobsson et al., bacterial contamination rates in home nebulizers of CF patients were examined. The findings indicated that contamination rates were generally low. However, individuals who did not follow the recommended disinfection and drying procedures were more likely to have infections. Significantly, none of the patients were colonized with the same contaminating organisms, suggesting that the contamination originated from the nebulizer device rather than the patients themselves [34]. 

As long as proper disinfection procedures are followed, people with CF can clean nebulizers and other respiratory equipment like spacers and airway clearance devices using tap water or well water that complies with local public health standards, distilled water, or bottled water. But only sterile water needs to be used for nasal rinses, filling humidifier reservoirs, and the last rinse for respiratory equipment following cold disinfection [29].

Vaccination

Vaccination plays a crucial role in preventing infections among individuals with CF. It is essential to adhere to recommended vaccination schedules and ensure that immunizations are kept up to date. The following section will cover the essential aspects [35].

Immune prophylaxis

Cystic fibrosis immune prophylaxis (CFIP) aims to enhance the immune system of individuals with CF to prevent or reduce the occurrence and severity of infections. The goal is to develop strategies that enhance innate and adaptive immune responses, enabling the body to combat the invaders effectively. Here, we will explore the essential methods to support the immune system of individuals who are suffering from CF.

Vitamin D

In vitro and clinical investigations have demonstrated the significant role of vitamin D in the innate immune system [35]. CF patients often experience vitamin D insufficiency or deficiency due to fat malabsorption caused by exocrine pancreatic insufficiency and inadequate dietary intake [36,37]. A randomized controlled trial conducted by Pincikova et al. showed that vitamin D treatment in children was associated with reduced levels of IL-8 in the blood, a marker of inflammation [38]. Additionally, several studies have indicated that vitamin D deficiency increases the risk of pulmonary exacerbation in children and adults with CF [39,40].

*Hypertonic Saline* 

Treatment with 7% hypertonic saline inhalation has been recognized as a cost-effective and productive method for preventing pulmonary exacerbations and enhancing the quality of life in individuals with CF [41]. The mechanisms by which hypertonic saline exerts its effects in CF can be explained as follows: a) alteration of mucus characteristics: hypertonic saline helps modify the properties of the mucus in the airways, making it less dense and easier to clear. This promotes mucus clearance and reduces airway obstruction; b) restoration of airway surface fluids: the inhalation of hypertonic saline aids in the rehydration of the airway surface, which can become dehydrated in individuals with CF. This rehydration helps improve the functioning of the respiratory epithelium and facilitates mucociliary clearance; c) stimulation of cough: hypertonic saline can stimulate the cough reflex, leading to mucus clearance and potentially reducing the risk of respiratory infections. These mechanisms collectively contribute to the beneficial effects of hypertonic saline inhalation in individuals with CF [42].

Prophylactic Antibiotics

Antibiotics have been widely used to prevent and treat infectious exacerbations in CF. There has been ongoing discussion regarding the role of prophylactic antibiotics in preventing infection or colonization. In cases where patients are colonized with Staphylococcus, some healthcare practitioners prescribe oral anti-staphylococcal antibiotics like flucloxacillin. The continuous use of prophylactic flucloxacillin in such situations has been associated with improved clinical outcomes, particularly during the first two years of life [43].

Early and aggressive antibiotic therapy has shown potential in preventing colonization and persistent *P. aeruginosa* infections [44,45]. Initiating anti-staphylococcal antibiotic prophylaxis in infancy and maintaining it until age six has been found to reduce the number of children with isolates of *Staphylococcus aureus* [46]. 

Vaccination for CF patients

In children and adults, wheezing or coughing is no reason to withhold or delay vaccination. Vaccinations should be withheld only under exceptional circumstances [47]. Here we are going to discuss the most critical vaccinations in CF patients.

Hepatitis A and B

Due to the absence of a functioning CF transmembrane conductance regulator protein on the biliary epithelium, individuals with CF are more susceptible to liver disease [48]. In light of this vulnerability, using a combination vaccine for hepatitis A and B in CF patients is worth considering. This combined vaccine has proven safe, well-tolerated, and highly immunogenic. It offers dual protection against hepatitis A and B viruses and can be administered following a simple schedule of doses at zero, one, and six months [49].

Measles-Mumps-Rubella Vaccine

The measles-mumps-rubella (MMR) vaccine has been a part of basic immunization regimens since the early 1980s, combining vaccinations against measles, rubella, and mumps. The initial dose of MMR vaccine is recommended to be administered at 12-15 months of age. Ensuring immunity to measles is particularly important for individuals with CF [50]. In many countries, CF adults may have received only one dose of the measles vaccine in the early 1980s before the subsequent guidelines recommended a booster dose during school age. Therefore, all CF adults who lack valid documentation of prior measles infection or received a complete and accurate immunization record after 12 months, consisting of two doses spaced at least four weeks apart, should receive the measles vaccine [50].

*Varicella* 

CF patients may be considered a target varicella-zoster (VZ) vaccination population for several reasons. First, VZ infection can contribute to the deterioration of lung function in CF patients. Second, a subset of the CF population, particularly those who have undergone transplantation, may require treatment with steroids or experience immunosuppression [51,52]. Regarding vaccination recommendations, children with CF should receive the VZ vaccine as soon as they are diagnosed, with a single dose administered between the ages of 1 and 13. Adolescents aged 13 and above, as well as young adults, who have not had a history of varicella infection, should receive two doses of the vaccine. These doses should be given four to eight weeks apart. There is no need for a third dose, even if the inter-dosage interval is longer [52].

Influenza

Among children who suffer from CF, influenza infection significantly increases the risk of hospitalization and respiratory complications. It has been recognized that influenza virus infection is one of the primary triggers for developing chronic *P. aeruginosa* infection in individuals with CF [53]. Therefore, vaccination against influenza is strongly recommended starting at the age of six months. Annual vaccination is considered the most effective approach to preventing influenza infection. It is now highly recommended that anyone above the age of six months at a higher risk of influenza complications due to age or medical condition receive annual influenza vaccination [54].

Pharmacological management

The cornerstone of daily CF treatment involves pancreatic enzyme replacement therapy (PERT), airway clearance therapies, and antimicrobial treatments [55]. 

Pancreatic Enzyme Replacement Therapy

Pancreatic insufficiency in CF patients, affecting approximately 85% of individuals, results in maldigestion and malabsorption. Early initiation of universal NBS, PERT, and nutritional intervention are paramount to improving clinical outcomes in CF infants. PERT has been found effective in improving clinical parameters and reducing gastrointestinal symptoms in patients with chronic pancreatitis, with enteric-coated microspheres (ECM) showing superior outcomes compared to enteric-coated tablets (ECT) in CF patients [56]. 

Airway Clearance Therapies

Mucociliary clearance therapies, including nebulized saline, mannitol, and rhDNase, are crucial in managing lung mucus viscosity alongside physiotherapy and exercise [55]. Regular use of nebulized hypertonic saline in adults and children over 12 with CF can improve lung function after four weeks, although this improvement may not be sustained at 48 weeks [41]. The evidence for its effectiveness in reducing pulmonary exacerbations and enhancing the quality of life in adults is inconclusive due to the varying quality of the studies. Additionally, in children rhDNase may offer better lung function improvement at three months compared to hypertonic saline, but the evidence for this is limited and inconclusive [41]. 

Antimicrobial Treatments

The high prevalence of pulmonary infections with resistant pathogens poses a significant challenge, highlighting the need for evidence-based guidelines for antibiotic treatment and the development of new therapies to disrupt biofilms [55]. Prospective cohort studies consistently show that intravenous antibiotic treatment improves lung function in CF patients with pulmonary exacerbations. However, some patients may have a suboptimal response, and higher inflammatory marker levels at exacerbation indicate a greater risk of non-response [57]. While symptoms improve during treatment, they may not reflect long-term lung function or the duration until subsequent antibiotic therapy [57].

The Trial of Optimal Therapy for Pseudomonas Eradication in Cystic Fibrosis (TORPEDO-CF) trial found no superiority of intravenous antibiotic treatment over oral treatment for eradicating* P. aeruginosa* in CF patients at early stages [58]. Although fewer patients in the intravenous group were admitted to the hospital in the 12 months after completion of the eradication regimen, there was no significant difference in the primary outcome of eradication rates or health-related quality of life between the two groups, suggesting that oral therapy is a more cost-effective and preferable option for early *P. aeruginosa *infection in CF [58]. The management of pulmonary exacerbations in CF remains challenging due to insufficient evidence and lack of consensus on various aspects of treatment decisions, including antibiotic selection, duration of intravenous antibiotic therapy, and the role of adjunctive therapies [57]. *P. aeruginosa *is a common bacterial pathogen that affects 55% of adult patients and can be eradicated by inhaled antibiotics in about 80% of cases. Recent research indicates that *P. aeruginosa *effects might not be completely mitigated by early eradication. Chronic mucoid *P. aeruginosa *infections in CF patients are difficult to eradicate, necessitating routine therapy with inhaled tobramycin every two months. Tobramycin resistance and deteriorating lung function advantages over time are causes for concern [3].

Antimicrobial Resistance in Cystic Fibrosis Respiratory Infections

The adaptation of pathogens in the CF lung environment leads to persistent infections, which involve various adaptive mechanisms, including the evolution of antimicrobial resistance (AMR) phenotype, biofilm formation, and the survival of persister subpopulations, making traditional antimicrobial susceptibility testing (AST), and resistance testing less clinically valuable, highlighting the need for alternative approaches such as investigating concentration values related to biofilm and defining clinical breakpoints for inhaled antibiotics, as well as exploring unconventional therapies like bacteriophages and antibiotic potentiating drugs in the limited drug development pipeline for anti-infective therapeutics [6,59]. 

*Cystic Fibrosis Transmembrane Regulator Modulators* 

The primary defect in CF is the presence of a mutant CFTR gene, which leads to decreased or absent CFTR protein activity, affecting chloride and bicarbonate transport in epithelial cells [60]. A total of more than 1500 mutations have been found in the CFTR gene, most commonly a deletion of phenylalanine at position 508 within class II mutations. Understanding CFTR dysfunction has paved the way for the development of pharmacologic compounds, including enhancers and correctors, that target specific abnormalities in CFTR function, offering potential therapeutic options for CF patients [60]. 

CFTR modulators are categorized into different groups based on their mechanisms of action, including enhancers that improve CFTR function, correctors that enhance CFTR expression and trafficking, amplifiers that stimulate CFTR protein expression, and stabilizers that rectify protein instability and increase CFTR membrane presence [1]. Examples of specific modulators include VX-770 (ivacaftor), which improves ion transport and is authorized for particular CF-causing variants, VX-809 (lumacaftor) used in combination with VX-770, VX-661 (tezacaftor) combined with VX-770, and elexacaftor in double or triple combinations, which have shown promising results in improving CFTR function [1]. Triple CFTR therapy consisting of two correctors (elexacaftor (ELX), tezacaftor (TEZ)) and a potentiator (ivacaftor (IVA)) acts by directly targeting the mutant CFTR channel-forming protein, interacting with the mutant F508del-CFTR polypeptide has shown promising results in improving the day-to-day activities and overall quality of life for CF patients, reducing symptoms such as shortness of breath, pulmonary exacerbations, and coughing, while also increasing energy levels and appetite [60]. The elexacaftor-tezacaftor-ivacaftor combination significantly improved pulmonary function, nutritional status, and overall quality of life for patients with advanced CF, potentially delaying lung transplantation. Real-world studies and patient perspectives provide promising evidence of long-term disease modification and positive outcomes for CF patients [61].

The inhibition of ENaC, when combined with CFTR modulators, has the potential to act as a synergistic driving force for Cl− secretion via rescued mutant CFTR channels in airway epithelial cells, offering a promising avenue for enhancing airway surface hydration, mucociliary clearance, and overall pulmonary outcomes in CF patients and other muco-obstructive lung diseases. However, further evaluation through clinical trials is warranted [62]. The promising exploration of CFTR modulators has significantly benefited the majority of CF patients, addressing gating and trafficking defects of the mutated CFTR protein; however, around 10% of patients remain unresponsive to these modulators due to specific mutations or intolerances, making gene therapy a mutation-independent and attractive option for this group, mainly targeting life-threatening lung disease until gene therapy advances to treat multiple affected organs in CF [63]. 

Gene therapy

While current therapies address symptoms, the fundamental genetic cause of CF is being targeted via gene therapy, which holds promise as a possibly permanent solution; nevertheless, difficulties with delivery strategies, immunological reactions, and the durability of modified cells must be resolved [64]. Due to the complexity of small compounds and the possibility of genotype-specific efficacy, there is considerable interest in creating gene therapies, particularly mRNA-based therapies, to treat CF. One method includes replacing the CFTR protein with mRNA, which is effective for all CF patients regardless of their specific mutation. In contrast, another system utilizes mRNA encoding for CRISPR-based gene editors to edit the genomic DNA in target cells, although it requires precise targeting of the mutation site. Both methods have shown promising results in animal models, with improved pulmonary function and ion conductivity observed upon delivery of exogenous CFTR mRNA using nanoparticles [65]. Using exon-skipping antisense oligonucleotide (ASO) therapy presents a promising approach for treating CF patients with the CFTR-W1282X nonsense mutation, a severe form of CF. By targeting the splice sites and promoting partially active CFTR protein expression, this strategy could enhance the function of CFTR and address the therapeutic needs of this specific patient population [66].

Lung transplantation

Despite additional challenges such as diabetes mellitus, nutritional issues, and colonization with drug-resistant bacteria, CF patients derive evident benefits from lung transplantation. The current lung allocation system has contributed to a more equitable distribution of organs, prioritizing individuals most likely to experience positive outcomes. Expanding efforts to enhance the donor pool and improve the lung function of potential donors should be a continued focus to further optimize transplantation outcomes in CF patients [67]. Many CF patients, who ultimately succumb to lung conditions, could potentially have increased their survival chances through lung transplantation. However, evidence indicates that the majority of these patients, who eventually die from respiratory diseases, have never been referred or considered for transplant, suggesting that some deaths might have been preventable with timely referrals [68]. 

Education

CF is a challenging and potentially life-threatening disease that necessitates a comprehensive understanding to manage its symptoms effectively [69]. In the 1950s, children born with CF had a significantly low survival rate, with few surviving beyond their first year of schooling. However, the median age for CF in the United States is 36.5 years [70]. Parents of children with CF often lack essential knowledge, resulting in unintentional poor adherence to treatment and inadequate transition of responsibility from parents to adolescents [69]. One study showed a notable disparity in disease-specific knowledge among different age groups, with children aged zero to six and seven to ten years exhibiting significantly higher learning than those in the 11-14 and 15-18 age groups [71]. All patients had low general medical and genetics/reproduction knowledge. Patients and parents heavily relied on doctors for information about CF, accounting for 77% of their knowledge source [71].

Clinic education can incorporate more comprehensive discussions on the reproductive implications of CF and the significance of mental health for overall well-being [72]. It would be beneficial to develop a systematic approach to ensure that these topics are covered by the early adolescent period. Healthcare providers can collaborate with patients to set educational milestones and adapt them based on the patient's preferences and expectations [72]. However, numerous individuals with CF and their caregivers actively participate in research initiatives facilitated by patient advocacy organizations like the CFF and Cystic Fibrosis Research, Inc. (CFRI). The CFF provides training and support to new patient partners and family members who engage in clinical quality improvement endeavors [73]. Within organizations like the Cystic Fibrosis Research and Education Patient Taskforce (CFReSHC), individuals with CF generate research proposal ideas and collaborate as equal partners with academics, stakeholders, and healthcare providers on research teams [73]. The CFReSHC members are willing and well-positioned to offer coaching and guidance to research teams new to Patient-Centered Outcomes Research (PCOR) when necessary [73].

Future educational models should go beyond providing basic information about the disease and incorporate discussions about its impact on daily life and mental health [72]. It is also crucial to acknowledge that caregivers differed in their reported areas of decreased confidence, particularly in novel therapies and other CF-related complications. Therefore, instructional programs must also consider caregivers' unique needs and concerns [72]. Failure to address the misconceptions, gaps, and errors in CF knowledge identified in the study may lead to unintended non-adherence to treatment and adversely affect the progression and outcome of the disease. It is necessary to develop educational programs tailored to the needs of chronically ill patients and their families [71].

Support 

In recent decades, the patient's role in CF care has evolved from passive recipient to active participant, especially with the burden of chronic disease and fragmented healthcare systems, pushing for patient engagement, communication through information and communication systems, and remote monitoring to enhance care coordination and improve treatment adherence [74]. The use of patient narratives in the form of "talking heads" videos and the positive deviance approach showed promise in inspiring confidence, promoting behavior change, and providing valuable insights into overcoming barriers to treatment adherence for individuals with CF [75]. Successful integration of an Advanced Healthcare Assistant (AHA) within an adult CF center, resulting in changes to physiotherapy service provision, increased complexity of clinical care, expanded physiotherapist roles, improved patient communication, and no reported safety issues, emphasizing the potential of AHAs to enhance service delivery in other acute respiratory physiotherapy settings [76]. 

Implementing a web-based platform that enables activity tracking, self-monitoring, and goal setting for CF patients has the potential to improve clinical outcomes, reduce healthcare utilization, and mitigate the financial burden associated with CF by promoting regular physical exercise as a low-cost treatment strategy and addressing challenges related to therapy delivery and adherence [77]. Living with the burdens of a chronic illness like CF brings forth not only physical challenges but also a sense of isolation, leading to a higher vulnerability for mental health struggles, with approximately 15% of individuals with CF reporting anxiety or depression and a staggering 41% experiencing both; thus, it is crucial to conduct clinical assessments for depression and anxiety in children aged seven to eleven when caregivers observe elevated symptoms or express concern, while annual screenings using tools like PHQ-9 and GAD-7 should begin at age 12 for individuals with CF and extend to caregivers as well [78]. 

## Conclusions

CF management has witnessed significant progress, offering hope and improved outcomes for patients with this complex genetic disorder. More efficient nebulized saline treatments and airway clearance therapies have been made possible by advancements in respiratory care, improving the overall quality of life and lung function. The domain of physiotherapy interventions is showing promise for strengthening respiratory function and reducing pulmonary exacerbations. However, research gaps persist in exercise interventions for CF patients, urging further exploration. Infection prevention strategies, such as strict hand hygiene and respiratory etiquette, are extremely important in lowering the incidence of respiratory infections in patients with CF. Pharmacological management has been revolutionized with the introduction of CFTR modulators, and gene therapy holds the promise of a transformative solution. Still, challenges surrounding delivery methods and immune responses necessitate further exploration. Patient education, support, and mental health assessments are essential for optimizing treatment adherence and addressing the emotional challenges of chronic illness. As research continues to unfold and medical science continues to advance with collaborative efforts between healthcare professionals, patient advocates, and researchers, the future looks promising in providing improved outcomes and brighter prospects for those affected by this challenging condition.
